# Supplementary material for: An Examination of the Anti-Cancer Properties of Plant Cannabinoids in Preclinical Models of Mesothelioma
Source: Cancers (Basel). 2022 Aug 5;14(15):3813. doi: 10.3390/cancers14153813 (PMC9367527; doi:10.3390/cancers14153813)
Supplement: Supplementary file 1 [file cancers-14-03813-s001.zip › cancers-1808569-Supplementary Figures.pdf]

cancers

MDPI

Supplementary Materials

An Examination of the Anti-Cancer Properties of Plant Cannabinoids in Preclinical Models of Mesothelioma

Emily K. Colvin, Amanda L. Hudson *, Lyndsey L. Anderson, Ramyashree Prasanna Kumar, Iain S. McGregor, Viive M. Howell and Jonathon C. Arnold*

Supplementary Methods

Oxidative stress assay: Cells were seeded and treated with the phytocannabinoid or vehicle control for 24 hours as described for the Cell Cycle Assay. After treatment, cells were harvested, stained, and analysed using the MUSE® Oxidative Stress Kit and the MUSE® Cell Analyser following the manufacturer's instructions.

Autophagy assay: Cells were seeded and treated with the phytocannabinoid or vehicle control for 24 hours as described for the Cell Cycle Assay. After treatment, cells were harvested, stained, and analysed using the MUSE® Autophagy LC3-Antibody based Kit and the MUSE® Cell Analyser following the manufacturer's instructions.

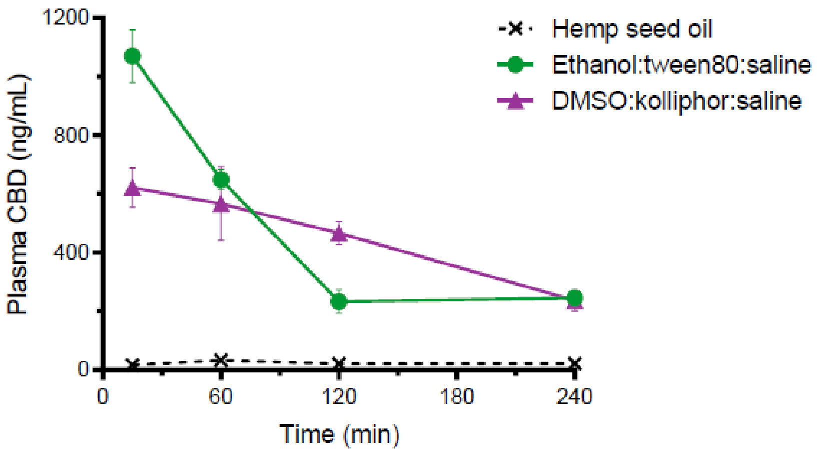

Figure S1. Pharmacokinetics of CBD using three standard vehicles. Rats were intraperitoneally injectedwith 20 mg/kg made up in hemp seed oil, ethanol:tween80:saline (1:1:18 ratio) or DMSO:kolliphor:saline (1:1:18 ratio). Blood was taken at 15 min, 1 h, 2 h and 4 h post injection and then analysed using LC-MS/MS.

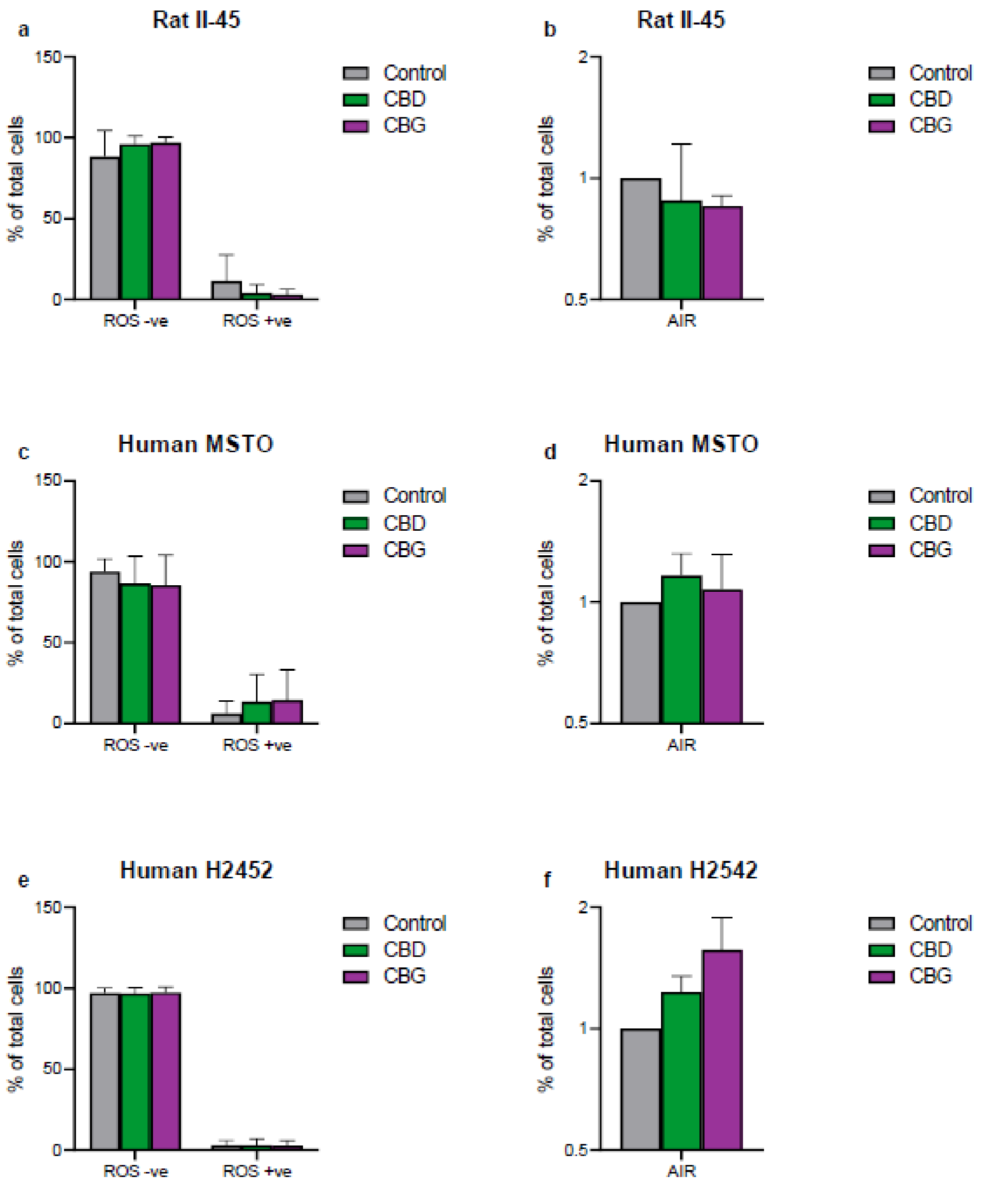

Figure S2. The phytocannabinoids CBD and CBG do not induce reactive oxygen species or autophagy in cultured mesothelioma cancer cell lines. The effects of CBD and CBG (2XIC50) on reactive oxygen species production and autophagy were assessed in rat II-45 (**a** and **b**), human MSTO (**c** and **d**), and H2452 (**e** and **f**) mesothelioma cells. After 24 h of treatment with vehicle (control), CBD or CBG, cells were harvested, and assays performed. Bars show the mean and SD from at least two independent experiments. No production of reactive oxygen species (ROS -ve); production of reactive oxygen species (ROS +ve); Autophagy induction ratio (AIR). No significance differences were identified using two-way ANOVA Holm-Sidak's multiple comparisons.
